# Comparison of Protective Effects of Electroacupuncture at ST 36 and LU 5 on Pulmonary and Hypothalamic Pituitary Adrenal Axis Changes in Perinatal Nicotine-Exposed Rats

**DOI:** 10.1155/2020/3901528

**Published:** 2020-01-19

**Authors:** Yawen Lu, Bo Ji, Guozhen Zhao, Jian Dai, Reiko Sakurai, Yitian Liu, Qiujie Mou, Yana Xie, Qin Zhang, Shuang Xu, Virender Kumar Rehan

**Affiliations:** ^1^School of Acupuncture-Moxibustion and Tuina, Beijing University of Chinese Medicine, Beijing 100029, China; ^2^Department of Pediatrics, Lundquist Institute for Biomedical Innovation at Harbor-UCLA Medical Center, Torrance, CA 90502, USA

## Abstract

**Background:**

Maternal smoking and/or exposure to environmental tobacco smoke continue to be significant factors in fetal and childhood morbidity and are a serious public health issue worldwide. Nicotine passes through the placenta easily with minimal biotransformation, entering fetal circulation, where it results in many harmful effects on the developing offspring, especially on the developing respiratory system.

**Objectives:**

Recently, in a rat model, electroacupuncture (EA) at maternal acupoints ST 36 has been shown to block perinatal nicotine-induced pulmonary damage; however, the underlying mechanism and the specificity of ST 36 acupoints for this effect are unknown. Here, we tested the hypothesis that compared with EA at ST 36, EA at LU 5 acupoints, which are on lung-specific meridian, will be equally or more effective in preventing perinatal nicotine-induced pulmonary changes.

**Methods:**

Twenty-four pregnant rat dams were randomly divided into 4 groups: saline (“S”), nicotine (“N”), nicotine + ST 36 (N + ST 36), and nicotine + LU 5 (N + LU 5) groups. Nicotine (1 mg/kg, subcutaneously) and EA (at ST 36 or LU 5 acupoints, bilaterally) were administered from embryonic day 6 to postnatal day 21 once daily. The “S” group was injected saline. As needed, using ELISA, western analysis, q-RT-PCR, lung histopathology, maternal and offspring hypothalamic pituitary adrenal axes, offspring key lung developmental markers, and lung morphometry were determined.

**Results:**

With nicotine exposure, alveolar count decreased, but mean linear intercept and septal thickness increased. It also led to a decrease in pulmonary function and PPAR*γ* and an increase of *β*-catenin and glucocorticoid receptor expression in lung tissue and corticosterone in the serum of offspring rats. Electroacupuncture at ST 36 normalized all of these changes, whereas EA at LU 5 had no obvious effect.

**Conclusion:**

Electroacupuncture applied to ST 36 acupoints provided effective protection against perinatal nicotine-induced lung changes, whereas EA applied at LU 5 acupoints was ineffective, suggesting mechanistic specificity and HPA axis' involvement in mediating EA at ST 36 acupoints' effects in mitigating perinatal nicotine-induced pulmonary phenotype. This opens the possibility that other acupoints, besides ST 36, can have similar or even more robust beneficial effects on the developing lung against the harmful effect of perinatal nicotine exposure. The approach proposed by us is simple, cheap, quick, easy to administer, and is devoid of any significant side effects.

## 1. Introduction

The concept that smoking is harmful to health has been deeply rooted among the populace, but tobacco smoking continues to hurt, particularly, the most vulnerable members of the society, i.e., fetuses and newborns. Maternal smoking and/or exposure to environmental tobacco smoke continue to be significant factors in fetal and childhood morbidity and are a serious public health problem worldwide. According to the World Health Organization, more than 1 billion people around the world smoke, and 5 million die from cigarettes each year. In China, the current smoking rate for adults, aged 15 and over, is 27.7, percent and the total number of smokers is more than 300 million (China Adult Tobacco Survey, 2015). Even though the rate of active smoking during pregnancy in China is relatively low, the exposure rate to secondhand smoke during pregnancy is alarmingly high (>50%) [1]. Globally, the rate of smoking during pregnancy varies from 5% to 40% in European countries [2], and about 10% of women in the United States smoke during pregnancy [3]. Currently, many people choose e-cigarettes instead of traditional tobacco products, but the harm caused by nicotine is almost equivalent [4, 5].

There are hundreds of toxicants in tobacco smoke, among which nicotine is the main chemical with known toxicity for the developing lungs and brain [6, 7]. Nicotine easily crosses placenta with minimal biotransformation, and it enters fetal circulation, potentially exceeding 15% of maternal circulation levels. Nicotine levels in the amniotic fluid can exceed maternal plasma levels by 88% [8, 9]. Perinatal nicotine exposure is harmful to the health of the offspring in many aspects; it results in low birth weight, preterm delivery, stillbirth, neurobehavioral deficits, sudden infant death syndrome, and a range of neuroendocrine, craniofacial, and immune system abnormalities [10–16]. In addition to these problems, it is especially harmful to the developing respiratory system. Previous studies have shown lifelong and even transgenerational pulmonary structural and functional changes, consistent with asthma phenotype, among other respiratory morbidities [17].

Despite aforementioned concerns, at present, other than smoking cessation and avoiding cigarette smoke exposure altogether, there is no effective preventive or therapeutic option. Unfortunately, due to aggressive advertising strategies from the tobacco industry, especially towards the youth, and the highly addictive property of nicotine and negative reinforcement of withdrawal symptoms [18, 19], it is unlikely that the problem of smoke/nicotine exposure will go away anytime soon. Therefore, newer, effective approaches are needed to deal with a public health issue of huge clinical, financial, and societal implications.

While pharmacological interventions remain far-fetched, interestingly, perinatal electroacupuncture (EA) offers a promising, practical approach that can be, safely and relatively easily, translated to clinical practice. Using a rat model, we recently showed that EA at ST 36 acupoints (Zusanli) effectively blocked perinatal nicotine-induced pulmonary phenotype in the exposed offspring [20, 21]. However, the specificity of the acupoints ST 36 in blocking perinatal nicotine-induced pulmonary phenotype is not established.

Zusanli (ST 36) is a commonly used acupuncture modality, utilized mainly, for general well-being and gastrointestinal ailments, although it has been also shown to be effective against respiratory conditions such as allergic asthma [22], chronic obstructive pulmonary disease [23], lung injury [24], and pulmonary fibrosis [25]. ST 36 acupoint is located on the Stomach Meridian of Hand-Yangming. In contrast, Chize (LU 5) belongs to the Lung Meridian of Hand-Taiyin (LU), which connects with the lung. Ancient and modern literature studies confirm that LU 5 can treat pulmonary diseases. Although LU 5 and ST 36 belong to different meridians, located on corresponding locations on elbow and knee joints, respectively, both can treat lung diseases. The purpose of this study was to determine whether EA applied at LU 5 acupoints could replicate or surpass the protective effect of EA at ST 36 on perinatal nicotine-induced lung phenotype. By examining the specificity of the beneficial effects of EA at ST 36 acupoints on perinatal nicotine-induced lung phenotype, we hope to gain better mechanistic insights and advance acupuncture's clinical translation for its potential benefits. We hypothesized that compared with EA at ST 36, EA at LU 5 acupoints would be equally or more effective in preventing perinatal nicotine-induced pulmonary phenotype.

### 1.1. Experimental Equipment and Reagents

Nicotine bitartrate (99% purity) and pentobarbital (2%) were purchased from Sigma-Aldrich, USA. HAN's acupoint stimulation equipment (LH202H) was obtained from Beijing Huawei Industrial Development Co., Ltd., and Huatuo brand aseptic acupuncture needles (0.20 mm × 13 mm) were acquired from Suzhou Medical supplies Factory Co., Ltd. Pulmonary function studies were performed using AniRes2005 animal lung function analysis system.

### 1.2. Animal Model and Grouping

Twenty-four specific pathogen-free (SPF) Sprague Dawley female (11-weeks old, weighing 210 ± 20 g) and eight male (11-weeks old, weighing 240 ± 30 g) rats were purchased from Beijing Weitong Lihua Experimental Animal Technology Co., Ltd. (certificate no. SCXK (Beijing) 2006-0009). The experiments were performed in the barrier animal room of the SPF Animal Research Center of Beijing University of Chinese Medicine. Before the experiment, the animals were allowed to acclimatize for 72 h at room temperature (23 ± 1)°C in 55 ± 5% relative humidity and 12 : 12 h light:dark cycle. All animal procedures were conducted in accordance with the Guidelines for the Animal Care and Use advocated by the U. S. National Institutes of Health, as well as the “3R” principle of animal experimentation with reduction, substitution, and optimization. The animal protocol passed the Ethics Committee of Beijing University of Chinese Medicine.

**Figure 1:**
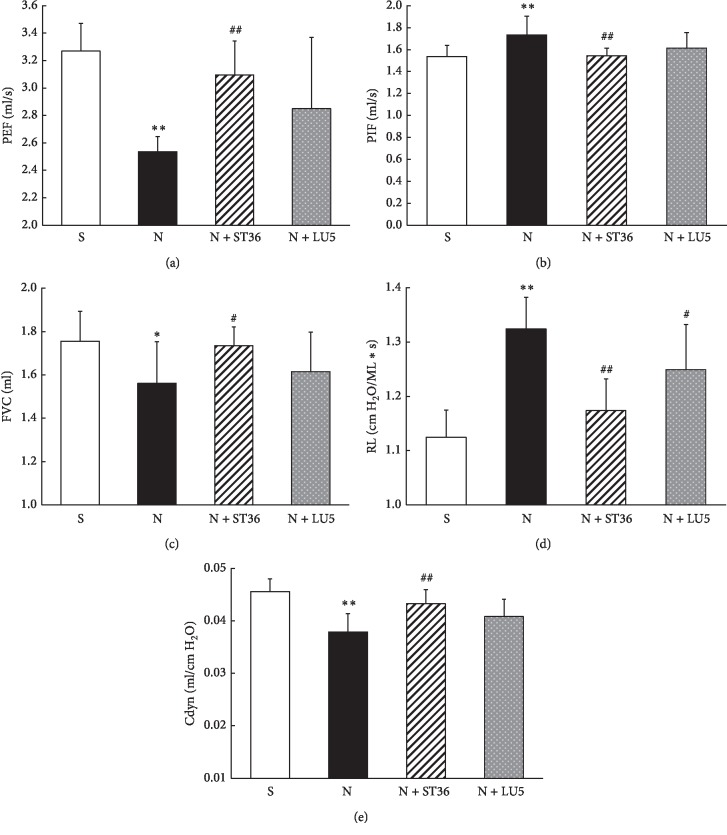
Effects of maternal EA on the lung function in perinatal nicotine exposure offspring (*n* = 8; ^*∗*^<0.05 vs. control; ^#^<0.05 vs. nicotine; ^*∗∗*^<0.01 vs. control; ^##^<0.01 vs. nicotine).

In line with our previously described model [26], pregnant dams were randomly divided to the saline (“S”), nicotine (“N”), nicotine plus Zusanli (N + ST 36), and nicotine plus LU 5 (N + LU 5) groups with 6 dams in each group. At term, dams delivered vaginally. The pups were culled to eight pups/litter and allowed to breast feed ad libitum. Nicotine (1 mg/kg/day∼moderate cigarette exposure) in 100 *µ*L volumes was administered to dams subcutaneously from embryonic day (*E*) 6 to postnatal day (PND) 21 except on the day of delivery. The “S” group was administered the same amount of saline subcutaneously as the “N” group from E6 to PND 21. The N + ST 36 group, in addition to nicotine, was administered acupuncture at acupoints ST 36, which are located in the posterolateral aspect of the knee joint, 5 mm below the fibulae capitulum (Figure 1). Similarly, the N + LU 5 group was administered acupuncture at LU 5 acupoints, which are located in the transverse cubital crease, on the radial side of the tendon of biceps brachii, i.e., located in the depression of the elbow transverse line on the radial side (Figure 1). The treatment method of EA was the same as that of N + ST 36 group.

For EA application, rat dams were held in a locally made restraining bag with head and legs outside of the bag at opposite ends, taped in prone position on a tabletop. When the dam was quiet, the Huatuo brand acupuncture needles (0.20 mm × 13 mm) were inserted perpendicularly 7 mm into ST 36 or LU 5 acupoints (connected to the EA negative electrode) and 2 mm subcutaneously below ST 36 or LU 5 (connected to the EA positive electrode). The EA parameters were set as follows: Sparse-dense wave frequency, 2/15 Hz; intensity, 1 mA; and duration, 20 minutes, administered by the same operator, once a day between 10 AM–12 noon every day.

### 1.3. Pulmonary Function Testing

For pulmonary function studies, on PND 21, after instrument calibration for airway pressure and lung volume, rat pups were weighed and anesthetized using 2% pentobarbital sodium (55 mg/kg), followed by tracheostomy, cannulation, and ventilation for plethysmography. After respiratory stability was achieved, using AniRes2005 software, airway resistance (RL), peak inspiratory flow (PIF), peak expiratory flow (PEF), forced vital capacity (FVC), and dynamic pulmonary compliance (Cdyn) were derived.

### 1.4. Sample Collection and Processing

#### 1.4.1. Lung Tissue Morphology

At sacrifice, the lungs were collected and instilled with ice-cold 4% paraformaldehyde (PFA) dissolved in 1 × PBS. These were kept immersed in 4% PFA for 4-5 hours and then moved to 30% sucrose dissolved in PBS. When settled to the bottom of the container, the lungs were removed, and the left lung was paraffin-embedded. An investigator unaware of experimental groups performed lung morphometry on 5 *μ*m thickness hematoxylin and eosin-stained tissue slices from different experimental groups. Using Image-Pro Plus 6.0 software, alveolar count, mean linear intercept, and septal thickness were determined as described by us previously [27].

#### 1.4.2. Determination of Lung PPAR*γ*, *β*-Catenin, Serum Corticosterone (Cort), and Pituitary Glucocorticoid Receptor (GR) Levels in Offspring and Pituitary Adrenocorticotropic Hormone (ACTH), Adrenal Melanocortin 2 Receptor (MC2R), and Serum Cort in Maternal Rats

At the end of the experimental period (PND 21), dams and pups were euthanized (pentobarbital 200 mg/kg, injected intraperitoneally), and blood was collected via cardiac puncture. Serum was separated by centrifugation, and samples were frozen at −80°C until processing. Offspring lungs and maternal pituitary and adrenal glands were collected and immediately flash-frozen in liquid nitrogen. Lung PPAR*γ* and *β*-catenin, serum Cort, and pituitary GR, ACTH, and adrenal MC2R levels were determined using ELISA according to manufacturers' instructions. As needed, the lung tissue, pituitary, and adrenal glands were ground, lysed, and centrifuged. ELISA kits used were PPAR*γ* from LifeSpan BioSciences Co., Ltd. (LS-F15392); *β*-catenin from Enzo Life Sciences Co., Ltd. (ADI-900-135); ACTH, MC2R, and GR from WUHAN CUSABIO Co., Ltd. (CSB-E06875r; CSB-E013559RA; and CSB-E08747r, respectively).

#### 1.4.3. Detection of PPAR*γ* and *β*-Catenin in Offspring Lung by Quantitative RT-PCR

RNA extraction was performed using Qiagen RNAeasy Mini kit (category no. 74106) obtained from Life Technologies. The extracted RNA was qualitatively analyzed using Agilent nucleic acid and protein analyzer (Agilent 2100, Germany) and quantified using Nanodrop micronucleic acid protein analyzer (UVS-99ASP-3700ASP, USA) and stored at −80°C until needed for assay. Reverse transcription of RNA to cDNA was performed using SuperScript VILOTM cDNA Synthesis Kit (category no. 11754) for qRT-PCR System (Life Technologies, USA) and the PCR automatic analyzer (PX2 Thermal, USA). Real-time PCR was performed using the upstream and downstream primers, obtained from Sangon Biotech Co., Ltd., Shanghai, China (Table 1). SYBR Green PCR Master Mix of PPAR*γ* and *β*-catenin were obtained from Invitrogen (USA) and amplified by the real-time fluorescence quantitative PCR (GeneAmp 7000 Sequence Detection System, Applied Biosystems). The reaction was carried out by activating the DNA polymerase at 95°C for 10 minutes, and then up to 40 PCR denaturation cycles at 95°C for 15 s and annealing at 60°C for 1 minute were performed. The relative quantitative value of each gene was calculated using the 2ΔΔCT method based on CT values.

#### 1.4.4. Statistical Analysis

All data were analyzed using the statistical software (IBM SPSS 20.0). The data are expressed as mean ± SD. The differences between the groups were compared by one-way analysis of variance, followed by the Tukey test. *P* < 0.05 indicated statistically significant difference.

## 2. Results

### 2.1. Effect of EA at ST 36 and LU 5 Acupoints of Perinatal Nicotine-Induced Pulmonary Phenotype in Offspring Rats

Compared with the “S” group, the PEF, FVC, and Cdyn in the “N” group decreased significantly (*P* < 0.01, *P* < 0.05, and *P* < 0.01, respectively), while the PIF and RL increased significantly (*P* < 0.01). Compared with the “N” group, the PEF, FVC, and Cdyn in the N + ST 36 group increased (*P* < 0.01, *P* < 0.05, and *P* < 0.01, respectively), and the PIF and RL decreased significantly (*P* < 0.01). Furthermore, compared with the “N” group, while the PEF, FVC, and Cdyn in the N + LU 5 group showed an upward and the RL, a downward trend; these changes did not reach statistical significance (*P* > 0.05) (Figure 1).

**Figure 2:**
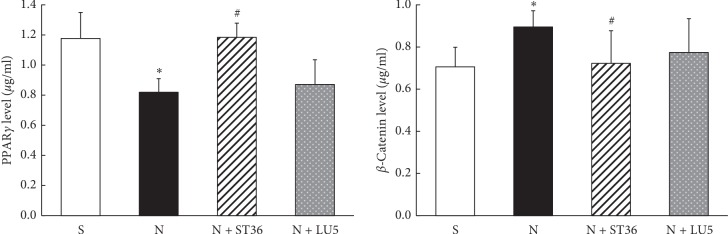
Effects of maternal EA on the levels of lung PPAR*γ* and *β*-catenin protein in PNE offspring (*n* = 6; ^*∗*^<0.05 vs. control; ^#^<0.05 vs. nicotine).

The results of ELISA showed that compared with the “S” group, PPAR*γ* protein level decreased, and *β*-catenin protein increased significantly in the “N” (*P* < 0.05). Compared with the “N” group, in the N + ST 36 group, PPAR*γ* protein level increased, and *β*-catenin levels decreased significantly (*P* < 0.05). However, in the N + LU 5 group, while there was a trend towards normalization, the changes did not statistical significance (*P* > 0.05) (Figure 2).

**Figure 3:**
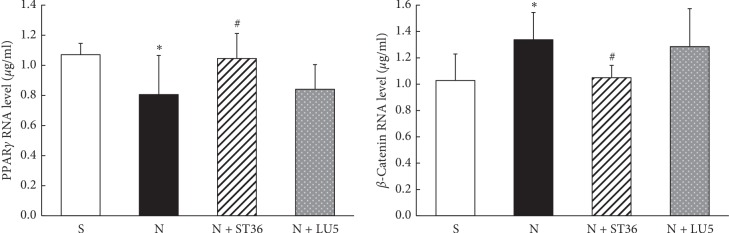
Effects of maternal EA on the lung PPAR*γ* and *β*-catenin mRNA in PNE offspring (*n* = 6; ^*∗*^<0.05 vs. control; ^#^<0.05 vs. nicotine).

The results of qRT-PCR for PPAR*γ* and *β*-catenin expression were in-line with the ELISA data outlined above, i.e., compared with the “S” group, “N” group showed a significant decrease in PPAR*γ* and a significant increase in *β*-catenin expression (*P* < 0.05). While these changes were corrected in the N + ST 36 group, there was no statistically significant effect in the LU 5 group (*P* > 0.05), even though there was a trend towards normalization (Figure 3).

### 2.2. Effect of EA at ST 36 and LU 5 Acupoints on Perinatal Nicotine-Induced Changes in Maternal HPA Axis

**Figure 4:**
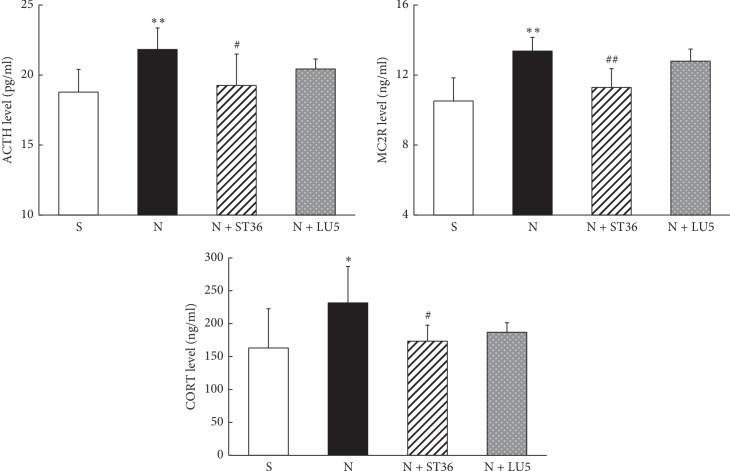
Effects of EA on PNE mother of maternal CORT and MC2R and ACTH (*n* = 6) (^*∗*^<0.05 vs control; ^#^<0.05 vs. nicotine; ^*∗∗*^<0.01 vs. control; ^##^<0.01 vs nicotine).

The results of ELISA showed that pituitary ACTH, adrenal MC2R, and serum Cort levels of rat dams in “N” group were significantly higher than that in “S” group (*P* < 0.01, *P* < 0.01, and *P* < 0.05, respectively). Compared with the “N” group, pituitary ACTH, adrenal MC2R, and serum Cort levels in N + ST 36 group were significantly lower (*P* < 0.05, *P* < 0.01, and *P* < 0.05, respectively) and not statistically different from the “S″ group; however, compared with the “N” group, there was no significant change in the levels of all these biomarkers in the N + LU 5 group (*P* > 0.05) (Figure 4).

### 2.3. Effect of EA at ST 36 and LU 5 Acupoints on Perinatal Nicotine-Induced Changes in Offspring HPA Axis

**Figure 5:**
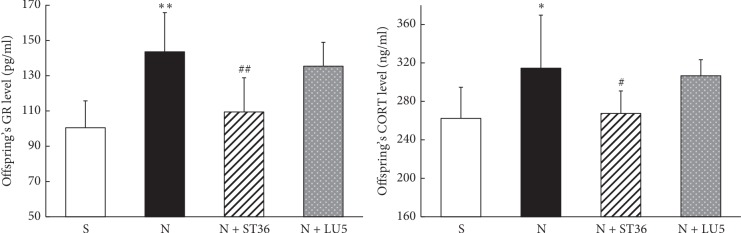
Effects of EA on PNE of offspring GR and CORT (*n* = 6; ^*∗*^<0.05 vs. control and ^#^<0.05 vs. nicotine; ^*∗∗*^<0.01 vs. control; ^##^<0.01 vs. nicotine).

Compared with the “S” group, lung GR and serum Cort levels in the offspring rats in the “N” group were significantly higher (*P* < 0.01, *P* < 0.05, respectively). The GR and Cort levels in the N + ST 36 group were significantly lower compared with the “N” group (*P* < 0.01 and *P* < 0.05, respectively) and were not statistically different from the “S” group (*P* > 0.05). In contrast, in the N + LU 5 group, while there was a downward trend, these levels were not statistically different compared with the “N” group (*P* > 0.05) (Figure 5).

### 2.4. Lung Morphometry

Compared with the “S” group, the mean linear intercept increased and alveolar count decreased significantly in the “N” group (*P* < 0.01 for both). Compared with the “N” group, these changes improved significantly in the N + ST 36 group (*P* < 0.05) and were not statistically different from the “S” group (*P* > 0.05). In contrast, in the N + LU 5 group, while there was an improving trend in these parameters, these did not reach statistical significance vs. the “N” group (*P* > 0.05). Note that lung morphometry data outlined here have been published previously [27].

## 3. Discussion

Our data demonstrate that EA at ST 36 blocked the perinatal nicotine-induced changes in maternal (hypothalamic CRH, adrenal MC2R, and serum ACTH and Cort levels) and offspring (serum Cort level and lung GR expression) HPA axes. It also blocked perinatal nicotine-induced changes in key lung developmental signaling pathways (PPAR*γ* downregulation and *β*-catenin upregulation), lung morphometry (radial alveolar count, mean linear intercept, and septal thickness), and pulmonary function (RL, PIF, PEF, FVC, and Cdyn). In contrast, EA at LU 5 acupoints had no significant effect on either maternal/fetal HPA axis, or lung molecular, structural, and functional phenotypes.

As outlined above, at present, there is no effective pharmacologic intervention against the harmful effects of perinatal smoke/nicotine exposure on the developing lung; however, our recent work has suggested acupuncture to be a promising, potential approach. Recently, we demonstrated that EA at ST 36 could regulate maternal HPA axis, promote PPAR*γ* and inhibit Wnt signaling in offspring lung, and preserve lung development and function in offspring perinatally exposed to nicotine [20, 21]. All of these findings have been reconfirmed in the present study, along with contrasting futility of EA at LU 5 in replicating these beneficial effects.

PPAR*γ* signaling is vital for lung lipid differentiation programming, which is essential for normal lung development and homeostasis [28]. PPAR*γ* signaling in turn interacts with Wnt signaling, which is involved in many key developmental biological processes involved in lung development and maturation [29–31]. A balanced PPAR*γ* and Wnt signaling is critically important during lung development [32], a balance that is clearly adversely impacted on perinatal smoke/nicotine exposure [33–36]. Additionally, the HPA axis regulates glucocorticoid levels, which also modulate pulmonary maturation and surfactant synthesis. Briefly, the hypothalamus secretes CRH, which regulates pituitary secretion of ACTH. ACTH regulates adrenocortical secretion of Cort, which, in turn, regulates the secretion of CRH via HPA axis feedback loop [37]. The maternal HPA axis promotes ACTH synthesis and cortisol release into the maternal circulation, which is transported across placenta. Under normal circumstances, the mother, placenta, and fetus work together to regulate the level of glucocorticoids in the uterine environment and prevent fetal exposure to excessive maternal glucocorticoids. Our results show that following perinatal nicotine exposure, maternal pituitary ACTH, adrenal MC2R, and serum Cort levels increase, indicating activated maternal HPA axis. Since nicotine has been shown to disrupt maternal-fetal placental barrier and reduce placental 11*β*-HSD-2 expression, it results in fetal exposure to a high glucocorticoid environment. This high fetal glucocorticoid environment potentially disrupts fetal neuroendocrine environment, inhibiting fetal HPA axis, intrauterine growth retardation [38], and elicitation of fetal compensatory responses, which, at least, partially explain a likely mechanism for how perinatal nicotine exposure might affect offspring lung development and how acupuncture might modulate it. In line with this paradigm, acupuncture has been shown to modulate HPA axis and regulate PPAR*γ* and *β*-catenin expressions [38–41]. In particular, EA at ST 36 blocked all perinatal nicotine-induced changes in maternal/offspring HPA axis in conjunction with the protected lung morphologic, molecular, and functional phenotype. In contrast, EA at LU 5 acupoints had no obvious effects on either maternal/offspring HPA axis or the resultant offspring lung phenotype. These results suggest that EA at ST 36 acupoints likely protects against the harmful effects of perinatal nicotine exposure on offspring lung development by primarily modulating maternal/offspring HPA axis, whereas EA at LU 5 acupoints had no such effect.

In the theory of traditional Chinese medicine, the main effects of acupoints' stimulation are directly related to the meridians and position to which they belong. The acupoints on the meridians can treat the diseases of the Zang-fu organs, which the meridians belong to, and the diseases of the Zang-fu organs and tissues, through which the meridians travel, as well as the disease of the adjacent areas. In addition, some acupoints have special therapeutic effects. The therapeutic effects of meridian acupoints are specific, an important factor affecting the beneficial effects of acupuncture. There are differences in functional effects between acupoints and nonacupoints, different acupoints of the same meridian, and different acupoints of different meridians. However, the specificity is relative, as one meridian point can treat multiple viscera and multisystem diseases, different meridian points can treat the same meridian or the same visceral diseases, and different meridian points have different effects on the same disease [42]. The two acupoints selected for this study belong to different meridians. ST 36 belongs to the Stomach Meridian Foot-Yangming (ST), which connects with the stomach. However, both ancient and modern literature supports that acupuncture at ST 36 has beneficial effects on not only gastrointestinal diseases but also on lung diseases. This is not surprising, since, embryologically, lung is derived from the foregut and maintains some functional commonality with gut, e.g., surfactant synthesis and secretion occurs in both organs. Moreover, in traditional Chinese medicine, invigoration of stomach and spleen in enhancing vital energy has been suggested to be the first important point of physical strength. Therefore, ST 36 has been often targeted to strengthen the physique and regulate the body as a whole. The recent work shows that ST 36 has a wide range of regulatory effects on various systems of the whole body, such as the neuroendocrine and immune systems [43]. In particular, by targeting neuroendocrine and immune mechanisms, it enhances the physique and self-repair and modulates various diseases. It is possible that the protective effect of ST 36 on lung development of offspring exposed to perinatal nicotine is related to its strong overall regulatory effect on whole body rather than a specific therapeutic effect on lung development per se. In contrast, since the LU 5 acupoints are connected to lungs, the main treatment targets of the acupoints on this meridian are related to the lung system. Chize (LU 5), the main point of the LU, can treat lung diseases. In addition, studies from ancient times to now have shown that although LU5 can also treat some nonlung diseases, e.g., diarrhoea and low back pain [44, 45]; there is very limited information on LU5 role in overall regulation of the whole body and effect commonly seen with ST 36 acupoints.

It is also important to highlight that in our previous studies, we did not observe any significant effects of EA at sham acupoints on lung morphometry and lung maturation supporting specific mechanisms involved in mediating ST 36 acupoints' effects on the developing lung. However, since we applied EA during both antenatal and postnatal periods, it is difficult to distinguish between the effects for each period, and to determine if postnatal intervention has any effect.

To sum up, comparing the effects of EA at ST 36 and LU 5 acupoints on lung development of the perinatally nicotine exposed offspring, we conclude that the mechanism underlying the beneficial effects of EA at ST 36 acupoints is via modulation of maternal/offspring HPA axis. This likely helps in avoiding offspring exposure to excessive maternal glucocorticoid environment induced by nicotine exposure, which, in turn, blocks the perinatal nicotine-induced decrease in alveolar PPAR*γ* and increase in Wnt signaling, and provides a more homeostatic milieu for offspring lung development. In contrast, even though LU 5 is on the lung meridian and has been used to treat many lung diseases, it did not prove to be useful against perinatal nicotine's exposure on the developing lungs. It reinforces that the beneficial effects of EA at ST 36 on the developing lung are via its effect on maternal/offspring HPA axis, rather than on offspring lung directly. This opens the possibility that other acupoints besides ST 36 having similar or even more robust beneficial effects on the developing lung against the harmful effect of perinatal nicotine exposure. The approach proposed by us is simple, cheap, quick, easy to administer, and is devoid of any significant side effects.

## Figures and Tables

**Table 1:** Primer sequence and length of amplification segment.

Gene name (rat)	Primer sequence (5′ to 3′)	Length of amplification segment
PPAR*γ*	Forward CCAAGTGACTCTGCTCAAGTATGG	106 bp
	Reverse CATGAATCCTTGTCCCTCTGATATG	
*β*-Catenin	Forward GTGCAATTCCTGAGCTGACC	184 bp
	Reverse CGGGCTGTTTCTACGTCATT	
GAPDH	Forward GACATGCCGCCTGGAGAAAC	92 bp
	Reverse AGCCCAGGATGCCCTTTAGT	

## Data Availability

The data used to support the findings of this study are available from the corresponding author upon request.

## References

[1] Zhang L., Hsia J., Xia X. (2015). Exposure to secondhand tobacco smoke and interventions among pregnant women in China: a systematic review. *Preventing Chronic Disease*.

[2] Smedberg J., Lupattelli A., Mårdby A.C. (2014). Characteristics of women who continue smoking during pregnancy: a cross-sectional study of pregnant women and new mothers in 15 European countries. *BMC Pregnancy & Childbirth*.

[3] Filion K., Abenhaim H., Mottillo S. (2011). The effect of smoking cessation counselling in pregnant women: a meta-analysis of randomised controlled trials. *BJOG: An International Journal of Obstetrics & Gynaecology*.

[4] Wagener T. L., Floyd E. L., Stepanov I. (2017). Have combustible cigarettes met their match? The nicotine delivery profiles and harmful constituent exposures of second-generation and third-generation electronic cigarette users. *Tobacco Control*.

[5] Etter J.-F., Bullen C. (2014). A longitudinal study of electronic cigarette users. *Addictive Behaviors*.

[6] Dempsey D. A., Benowitz N. L. (2001). Risks and benefits of nicotine to aid smoking cessation in pregnancy. *Drug Safety*.

[7] Eriksen M., Mackay J., Ross H. (2013). *The Tobacco Atlas*.

[8] Andres R. L., Day M. C. (2000). Perinatal complications associated with maternal tobacco use. *Seminars in Neonatology*.

[9] Benowitz N. L. (1998). *Nicotine Safety and Toxicity*.

[10] Vesterinen H. M., Morellofrosch R., Sen S. (2017). Cumulative effects of prenatal-exposure to exogenous chemicals and psychosocial stress on fetal growth: systematic-review of the human and animal evidence. *PLoS One*.

[11] Schechter J., Do E. K., Zhang J. J. (2018). Effect of prenatal smoke exposure on birth weight: the moderating role of maternal depressive symptoms. *Nicotine & Tobacco Research*.

[12] Dwyer J. B., Mcquown S. C., Leslie F. M. (2009). The dynamic effects of nicotine on the developing brain. *Pharmacology & Therapeutics*.

[13] Alkam T., Mamiya T., Yoshida N. (2017). Prenatal nicotine exposure decreases the release of dopamine in the medial frontal cortex and induces atomoxetine-responsive neurobehavioral deficits in mice. *Psychopharmacology*.

[14] Orzabal M. R., Lunde-Young E. R., Ramirez J. I. (2019). Chronic exposure to e-cig aerosols during early development causes vascular dysfunction and offspring growth deficits. *Translational Research*.

[15] Zhu C., Guo Y., Luo H. (2019). Synergistic effects of prenatal nicotine exposure and post-weaning high-fat diet on hypercholesterolaemia in rat offspring of different sexes. *Basic & Clinical Pharmacology & Toxicology*.

[16] Qu W., Zhao W.-h., Wen X. (2019). Prenatal nicotine exposure induces thymic hypoplasia in mice offspring from neonatal to adulthood. *Toxicology Letters*.

[17] Mcevoy C. T., Spindel E. R. (2016). Pulmonary effects of maternal smoking on the fetus and child: effects on lung development, respiratory morbidities, and Life long lung health. *Paediatric Respiratory Reviews*.

[18] Aguirre C. G., Bello M. S., Andrabi N. (2016). Gender, ethnicity, and their intersectionality in the prediction of smoking outcome expectancies in regular cigarette smokers. *Behavior Modification*.

[19] Rahmanian S. D., Diaz P. T., Wewers M. E. (2011). Tobacco use and cessation among women: Research and treatment-related issues. *Journal of Women’s Health*.

[20] Ji B., Zhao G.-Z., Cao R. (2016). Effect of maternal electroacupuncture on perinatal nicotine exposure-induced lung phenotype in offspring. *Lung*.

[21] Liu Y., Ji B., Zhao G. (2018). Protective effect of electro-acupuncture at maternal different points on perinatal nicotine exposure-induced pulmonary dysplasia in offspring based on HPA axis and signal transduction pathway. *Biochemical and Biophysical Research Communications*.

[22] Liang-liang C., An-sheng Li, Jian-ning T. (1996). Clinical and experimental studies on preventing and treating anaphylactic athma with Zusanli point immunotherapy. *Chinese Journal of Integrated Traditional and Western Medicine*.

[23] Xie J.-h., Yu J.-h. (2014). Effect of warming needle moxibustion on pulmonary function of elderly patients with stable chronic obstructive pulmonary disease. *World Journal of Acupuncture–Moxibustion*.

[24] Huang W., Zhang X., Liu Z. (2017). Effect of electroacupuncture pretreatment on inflammatory cytokines and aquaporin-5 in acute lung injury rats induced by lipopolysaccharide. *Journal of Yunnan University of Traditional Chinese Medicine*.

[25] Liu G., Liu S., Zhang C., LI F., LIU Y., Du Q. (2016). Clinical observation on the treatment of pulmonary fibrosis in rats through huangqi injection in acupoint Zusanli combined with shaoshang bloodletting. *Journal of Liaoning University of Traditional Chinese Medicine*.

[26] Sakurai R., Liu J., Gong M. (2016). Perinatal nicotine exposure induces myogenic differentiation, but not epithelial-mesenchymal transition in rat offspring lung. *Pediatric Pulmonology*.

[27] Yun-peng G. E., Bo J. I., Guo-zhen Z. H. A. O. (2019). Electroacupuncture at “Zusanli” (ST36) and “Chize” (LU5) of mother rats exposed to nicotine during pregnancy and lactation has a protective effect on development of lung function and morphology in neonatal rats. *Acupuncture Research*.

[28] Karadag A., Sakurai R., Wang Y. (2009). Effect of maternal food restriction on fetal rat lung lipid differentiation program. *Pediatric Pulmonology*.

[29] Guo M. K., Shao C., Wang J. (2016). Wnt/*β*-catenin signaling plays an ever-expanding role in stem cell self-renewal, tumorigenesis and cancer chemoresistance. *Genes & Diseases*.

[30] Clevers H., Loh K. M., Nusse R. (2014). An integral program for tissue renewal and regeneration: Wnt signaling and stem cell control. *Science*.

[31] Königshoff M., Eickelberg O. (2010). WNT signaling in lung disease. *American Journal of Respiratory Cell and Molecular Biology*.

[32] Torday J. S., Rehan V. K. (2006). Up-regulation of fetal rat lung parathyroid hormone-related protein gene regulatory network down-regulates the sonic hedgehog/wnt/βcatenin gene regulatory network. *Pediatric Research*.

[33] Rehan V. K., Asotra K., Torday J. S. (2009). The effects of smoking on the developing lung: insights from a biologic model for lung development, homeostasis, and repair. *Lung*.

[34] Mcevoy C. T., Schilling D., Clay N. (2014). Vitamin C supplementation for pregnant smoking women and pulmonary function in their newborn infants: a randomized clinical trial. *Jama*.

[35] Hoo A.-F., Henschen M., Dezateux C., Costeloe K., Stocks J. (1998). Respiratory function among preterm infants whose mothers smoked during pregnancy. *American Journal of Respiratory and Critical Care Medicine*.

[36] Hanrahan J. P., Tager I. B., Segal M. R. (1992). The effect of maternal smoking during pregnancy on early infant lung function. *American Review of Respiratory Disease*.

[37] Allen A., Schenkenberger I., Trivedi R. (2013). Inhaled fluticasone furoate/vilanterol does not affect hypothalamic-pituitary-adrenal axis function in adolescent and adult asthma: randomised, double-blind, placebo-controlled study. *The Clinical Respiratory Journal*.

[38] Jia B., Li Z., Shi Y. (2004). Effect of electroacupuncture on changes of behavior and some relat ed hormones of hypothalamus-pituitary-adrenal Axis in chronic stress model rats. *Acupuncture Research*.

[39] Cai Y., Liu Z., Wang S. (2009). Influence of electroacupuncture of meridian acupoints on the related hormones of the hypothalamus-pituitary-adrenal Axis in rats with cerebral ischemia reperfusion injury. *Acupuncture Research*.

[40] Jing X., Ou C., Chen H. (2016). Electroacupuncture reduces weight gain induced by rosiglitazone through PPAR*γ* and leptin receptor in CNS. *Evidence-Based Complementary and Alternative Medicine*.

[41] Liu J., Sakurai V. K. R., Rehan V. K. (2015). PPAR-*γ* agonist rosiglitazone reverses perinatal nicotine exposure-induced asthma in rat offspring. *American Journal of Physiology-Lung Cellular and Molecular Physiology*.

[42] Liang F., Zeng F., Zhao L. (2009). Specificity of acupoint effect and its fundamental laws. *World Journal of Acupuncture-Moxibustion*.

[43] Niu W., Niu X., Lei Z. (2014). Effect of acupuncture and moxibustion at Zusanli point on neuroendocrine immune network system. *Journal of Shaanxi University of Chinese Medicine*.

[44] Wang F. (2004). Treatment of 84 cases of acute tonsillitis by pricking blood on the back of the ear at LU5. *Journal of Clinical Acupuncture and Moxibustion*.

[45] Kong D. (2013). Discussion on the treatment of backache by using chize (LU5) point. *Modern Traditional Chinese Medicine*.

